# Composition of the Hemagglutinin Polybasic Proteolytic Cleavage Motif Mediates Variable Virulence of H7N7 Avian Influenza Viruses

**DOI:** 10.1038/srep39505

**Published:** 2016-12-22

**Authors:** E. M. Abdelwhab, Jutta Veits, Reiner Ulrich, Elisa Kasbohm, Jens P. Teifke, Thomas C. Mettenleiter

**Affiliations:** 1 Institute of Molecular Virology and Cell Biology, Friedrich-Loeffler-Institut, Federal Research Institute for Animal Health, Greifswald-Insel Riems, Germany; 2 Department of Experimental Animal Facilities and Biorisk Management, Friedrich-Loeffler-Institut, Federal Research Institute for Animal Health, Greifswald-Insel Riems, Germany; 3 Institute of Epidemiology, Friedrich-Loeffler-Institut, Federal Research Institute for Animal Health, Greifswald-Insel Riems, Germany

## Abstract

Acquisition of a polybasic cleavage site (pCS) in the hemagglutinin (HA) is a prerequisite for the shift of low pathogenic (LP) avian influenza virus (AIV) to the highly pathogenic (HP) form in chickens. Whereas presence of a pCS is required for high pathogenicity, less is known about the effect of composition of pCS on virulence of AIV particularly H7N7. Here, we investigated the virulence of four avian H7N7 viruses after insertion of different naturally occurring pCS from two HPAIV H7N7 (designated pCSGE and pCSUK) or from H7N1 (pCSIT). *In vitro*, the different pCS motifs modulated viral replication and the HA cleavability independent on the HA background. However, *in vivo*, the level of virulence conferred by the different pCS varied significantly. Within the respective viral backgrounds viruses with pCSIT and pCSGE were more virulent than those coding for pCSUK. The latter showed also the most restricted spread in inoculated birds. Besides the pCS, other gene segments modulated virulence of these H7N7 viruses. Together, the specific composition of the pCS significantly influences virulence of H7N7 viruses. Eurasian LPAIV H7N7 may shift to high pathogenicity after acquisition of “specific” pCS motifs and/or other gene segments from HPAIV.

The family *Orthomyxoviridae* encompasses six genera designated Influenzavirus A, B, C, Thogotovirus, Quaranjavirus and Isavirus^1^. Influenza A viruses are further divided into old and recent equine, classical swine/human, gull and avian influenza virus (AIV) lineages^2^,^3^ in addition to a recently discovered lineage of influenza A viruses found in bats^4^. AIV contain a single-stranded, negative sense ribonucleic acid (RNA) genome composed of eight gene segments; each segment is wrapped by the nucleoprotein and associates with the viral polymerase subunits in the ribonucleoprotein complex^2^,^3^. At least 10 viral proteins are expressed in host cells. Based on the variability of the envelope glycoproteins hemagglutinin (HA) and neuraminidase (NA), 16 different HA and 9 different NA subtypes had been described in AIV and isolated from birds in variable combinations^2^.

AIV of H5 and H7 subtypes vary in their virulence. In poultry, mass deaths are caused by highly pathogenic (HP) AIV subtypes which evolve from low pathogenic (LP) progenitors after circulation in domesticated birds for a few weeks up to several months^5^. Modulation of virulence from LP to HP in domestic poultry may depend on multiple genes in nature. However, HA is the major determinant of virulence^6^ which has been associated with alterations in the proteolytic cleavage site (CS) of the HA protein from a monobasic arginine (R) or lysine (K), to a polybasic CS (pCS) with a minimal ^−4^R-x-K/R-R^−1^/GLF motif^7^. This alteration results in HA cleavage-activation not only by tissue-restricted trypsin-like proteases that cleave LPAIV in the respiratory and digestive tracts, but also by ubiquitous furin-like proteases found throughout the host resulting in systemic infection^6^. Nevertheless, several H5 and H7 viruses with pCS exhibited relatively low virulence in chickens and further mutations within or beyond the HA were required for high virulence and/or efficient transmissibility in birds^8^,^9^,^10^,^11^,^12^,^13^,^14^. To date little is known about the impact of different pCS sequences on virulence of AIV in chickens and modulation of virulence by the reassortment of different gene segments. Moreover, in contrast to H5 viruses, there is a lack of knowledge on the genetic determinants for virulence and adaptation of recent H7 viruses in poultry particularly in Eurasia.

H7 viruses were frequently isolated in wild birds in Europe^15^. In the last decade, Europe has experienced several outbreaks of HPAIV H7N7 in domestic poultry upon transmission from wild birds^15^,^16^,^17^,^18^,^19^. The HPAIV H7N7 epidemic that occurred in poultry in the Netherlands from February to May 2003, and spread to poultry in Germany and Belgium^18^,^20^ was unprecedented. The closest ancestors of the HA and NA genes of this virus were H7N3 and H10N7 viruses that were isolated from mallard ducks in the Netherlands^17^,^20^,^21^. The putative LPAI mallard virus had a monobasic CS “PEIP**KG**R/GLF”, while the HPAIV H7N7 had a characteristic pCS “PEIP**KRRR**R/GLF”^20^. Unexpectedly, the Dutch HPAIV H7N7 infected humans representing one of the largest documented AIV-related human outbreaks^22^,^23^. Therefore, the virulence of the parental virus was extensively studied in mouse and ferret models^24^,^25^. However, the molecular basis for virulence in the original avian host (i.e. chickens) is not known. The most recent outbreak of HPAI H7N7 occurred in July 2015 in the United Kingdom (UK) (http://www.oie.int/en/animal-health-in-the-world/update-on-avian-influenza/2015/). This H7N7 virus specified a unique pCS “PEIP**RHRKG**R/GLF”.

In addition, several incursions of LPAI H7N7 into poultry were reported in Europe including Germany, where the source of infection was mostly linked to wild birds or contemporary outbreaks in poultry in the Netherlands^16^. These LPAIVs specify an identical monobasic CS motif PEIP**KG**R/GLF. The virulence potential of these LPAIVs remains undefined. We studied the importance of the pCS for the shift in virulence in chickens by inserting three different pCS into four different German H7N7 viruses; a HPAIV H7N7 isolated from chickens in 2003 (designated H03), and three different LPAIV H7N7 isolated from turkeys or chickens in 2001 (L01), 2011 (L11) and 2013 (L13). The different pCS motifs were found in naturally occurring HPAIVs during H7N7 outbreaks in the Netherlands, Belgium and Germany in 2003 (designated pCSGE), the recent British H7N7 outbreak in 2015 (designated pCSUK) and H7N1 outbreaks in Italy in 1999–2000 (designated pCSIT; PEIPKG**SRVR**R/GLF).

## Results

### H7N7 viruses in domestic poultry in Europe clustered in two different HA phylogroups with viruses from wild and domestic birds in Eurasia

**Figure 1:**
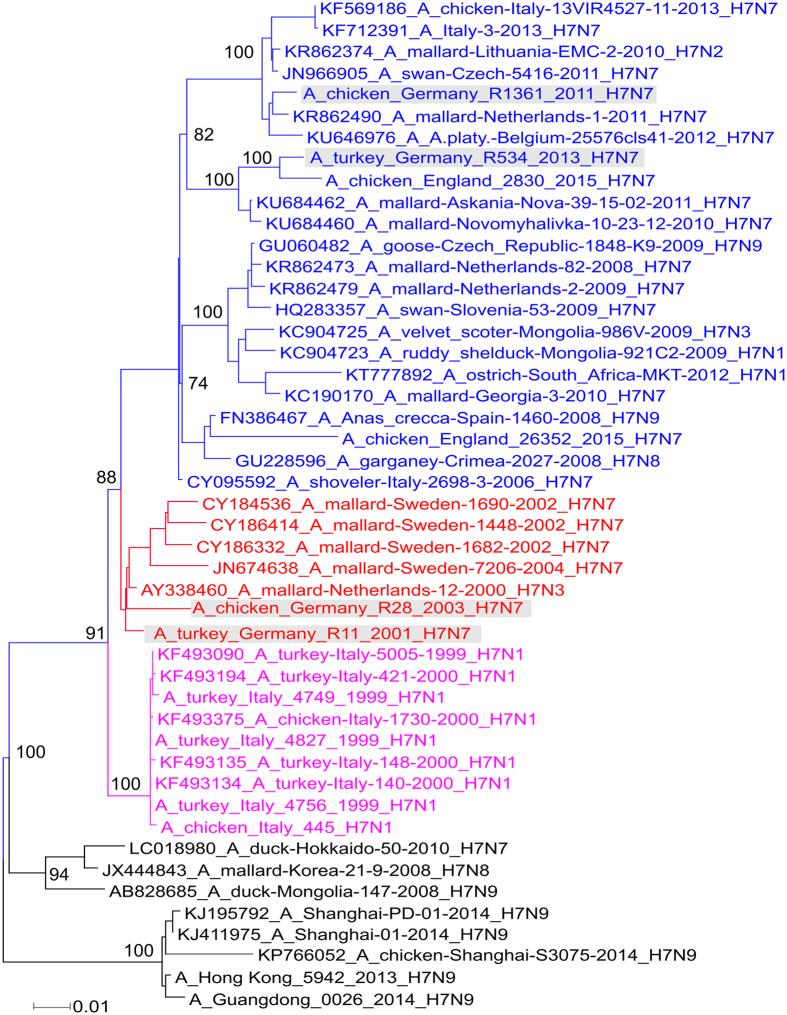
Phylogenetic relationship of the HA gene segment of Eurasian H7 viruses. HA gene sequences of all avian influenza H7 viruses from Europe, Asia and Africa were retrieved from GenBank and GISAID in addition to viruses generated in this study (marked in grey). Maximum likelihood midpoint rooted trees were constructed from selected sequences by MrBayes according to the best fit model using Topali v2^51^ and further edited using FigTree and Inkscape free software. European H7 viruses clustered in three phylogroups: group 1 (magenta), group 2 including L01 and H03 (red) and group 3 including L11 and L13 (blue).

To study the genetic relationship of the viruses used in this study to other Eurasian H7 AIV, HA gene sequences of all avian influenza H7 viruses from Europe, Asia and Africa were retrieved from GenBank and GISAID. Sequences generated in this study were also taken into account. A total of 1884 HA gene sequences (of at least 1600 nucleotides each) from H7 AIV were aligned using MAFFT, and phylogenetic trees were constructed using IQ-TREE after estimation of the best fit substitution models. Selected sequences were further analysed by MrBayes (Fig. 1). The HA showed clustering of the European H7 viruses in three distinct groups: the Italian H7N1/1999–2001 group (designated group 1), H7N3/N7/N9 from wild mallards in Sweden and the Netherlands from 2000 to 2010 (designated group 2), and H7 viruses (all NA subtype combinations except N5 and N6) from wild and domestic birds from several countries in Europe, Asia and Africa from 2002 to 2016 (designated group 3). L01 and H03 clustered in group 2, whereas L11 and L13 clustered in group 3 with the recent H7N7 virus in the UK (Fig. 1). Thus, the viruses selected for further study represent two different genetic groups.

### Generation of recombinant viruses and mutants

In order to assess the impact of pCS on virulence, we rescued four H7 AIVs: one HP (H03) and three LP (L01, L11 and L13). In addition, the pCS from three HPAIVs (including that from H03) was inserted to replace the pCS of H03 or to replace the CS of the three LPAIVs. Alternatively, the whole HA gene segment of L01 or L11 was replaced by the cognate segment from H03. In total, 19 distinct viruses were generated. Recombinant viruses generated in this study and their HA CS motifs are shown in Table 1. Viruses were arranged in the tables and figures according to the experimental settings *in vivo* and *in vitro*.

### Different polybasic proteolytic cleavage motifs conferred variable virulence of Eurasian H7N7 AIVs in chickens

**Figure 2:**
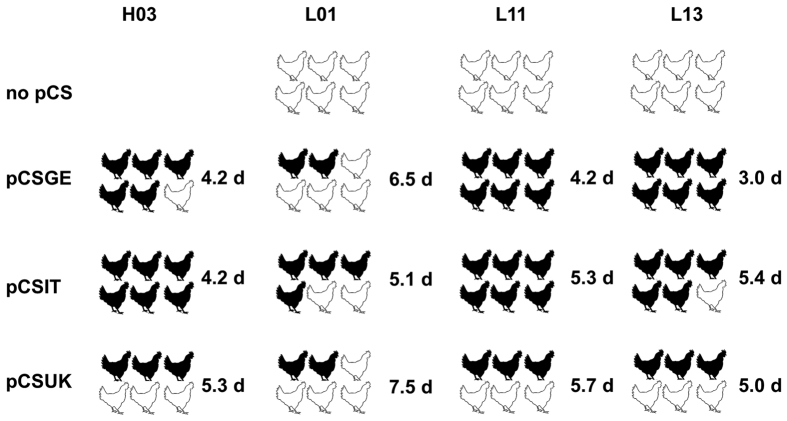
Impact of different cleavage site motifs on mortality and mean death time in inoculated chickens. Six chickens were inoculated with different recombinant viruses by oculonasal route. Black chickens refer to dead chickens after inoculation while white chickens refer to animals which survived the infection. Numbers refer to the mean death time (MDT) per day (d) after inoculation for 10 days observation period.

**Figure 3:**
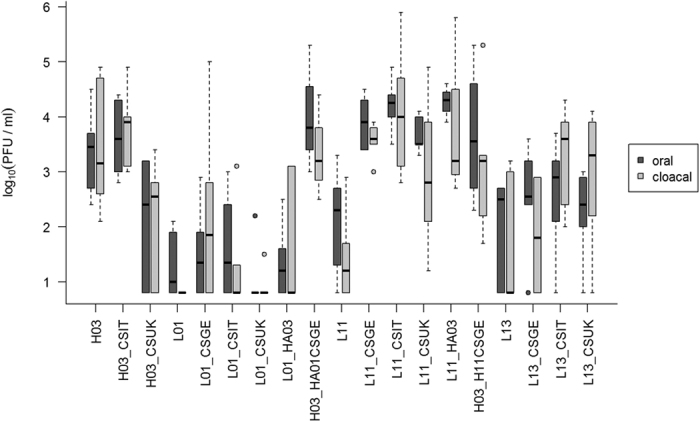
Virus excretion in swabs collected from inoculated birds. Viruses were arranged in the figure according to the experimental settings *in vivo* and *in vitro*. Virus excretion in oropharyngeal and cloacal swabs at 4 dpi was determined by RT-qPCR. All birds inoculated with L13_CSGE died at 3 dpi; therefore swabs collected at 2 dpi were examined. Relative quantity was expressed as PFU/ml and was calculated from standard curves with a ten-fold serial diluted H7N7 virus and a calculated detection limit of about 7 PFU/ml. The boxes depict the interquartile range (IQR, first to third quartile) including the median (horizontal bar). The whiskers extend to the most extreme value up to 1.5 times the IQR. Circles mark outliers that fall out of this range.

Six birds were inoculated oculonasally with each recombinant virus and 1 dpi 4 birds were added to assess virus transmission. Oropharyngeal and cloacal swabs were examined by RT-qPCR for relative quantitation of virus excretion. Tissues were collected from inoculated birds at 4dpi for histopathological examination. In contrast to HPAIV H03, LPAIV L01, L11 and L13 did not kill any inoculated or contact bird, and induced no or only transient mild clinical signs (Table 2; Fig. 2). The amount of virus excretion as well as the amount of antigen in birds inoculated with L01 (and derivatives) was lower than in H03, L11 and L13 inoculated animals which we assume could result from insufficient adaptation to poultry (Fig. 3). Insertion of pCSGE, pCSIT or pCSUK increased the virulence and spread of all three LPAIVs in chickens, to an extent that varied depending on the sequence of the polybasic motif (Fig. 2). Transmission of these viruses was enhanced as seen by high morbidity, mortality and virus excretion in cagemates.

### pCSGE increased virulence, tropism and virus excretion of L11 and L13 more than in L01

**Figure 4:**
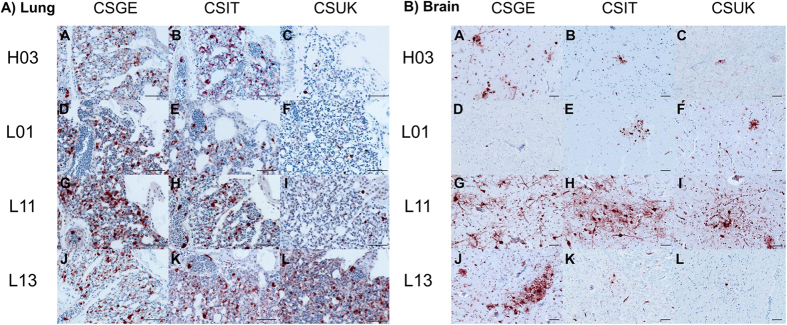
Impact of the different polybasic cleavage sites on organ distribution of AIV in the lung and brain. Shown is the organ distribution for each virus in the lung (**A**) and brain (**B**) of birds inoculated with H03 (A–C), L01 (D–F), L11 (G–I) or L13 (J–L). Influenza virus nucleoprotein was detected by immunohistochemistry (ABC method, intranuclear and intracytoplasmic red-brown antigen signal by 3-amino-9-ethyl-carbazol chromogen and hematoxylin (blue) counterstain). Bars = 50 μm.

H03 exhibited high virulence in chickens since 5 out of 6 inoculated birds died with a mean death time (MDT) of 4.2 days post-inoculation (dpi) and 3 out of 4 contact birds died within 7 days post-contact (dpc), respectively (Table 2). Surviving birds showed moderate to severe clinical signs until the end of the experiment, and all inoculated birds at 4 dpi and 2/4 contact birds at 3 dpc excreted virus in oropharyngeal and/or cloacal swabs (Fig. 3). The virus showed high tropism to the vascular endothelium of all organs examined, and was detected in all organs with high antigen concentrations in lungs and brain (Fig. 4).

Insertion of pCSGE into L01 resulted in a virus that killed only 2 out of 6 inoculated birds and 2 out of 4 contact birds with MDT of 6.5 days (Table 2). Like L01, L01_CSGE was detected in oropharyngeal swabs in 3/6 inoculated birds only. In cloacal swabs, none of the birds shed L01 virus but 4 out of 6 birds inoculated with L01_CSGE were positive. Viral RNA was rarely detected in contact birds inoculated with L01 and recombinants at 3 dpc (data not shown). In contrast to L01, L01_CSGE antigen was detected in the vascular endothelial cells of all organs.

All birds inoculated with L11_CSGE died with MDT of 4.2 days and all contacts died within 8 dpc (Table 2). L11 RNA was detected in 5/6 inoculated and 1/4 contact chickens, but L11_CSGE was detected at significantly higher levels from all inoculated and contact birds. While L11 was detected only in the vascular endothelium in spleen and bursa of Fabricius, L11_CSGE was detectable in multiple organs at high antigen content.

L13_CSGE killed all inoculated birds with MDT of 3 days and all contact birds died within 5 dpc (Table 2). Comparable levels of RNA were detected in birds inoculated with L13 (n = 4/6) and L13_CSGE (n = 5/6) and their contact cagemates (n = 1/4 and 2/4), respectively. L13 antigen was not detected in any of the organs examined, although histopathological changes were observed in the lung, pancreas, liver, gastrointestinal tract and brain (data not shown). Conversely, high antigen amount of L13_CSGE was detected by immunohistochemical examination in vascular endothelial cells as well as multiple organs.

### Like pCSGE, insertion of pCSIT conferred higher virulence to H03, L11 and L13 than to L01

H03_CSIT killed all inoculated chickens (n = 6) with MDT of 4.2 days and contact birds (n = 4) by 7 dpi (6 dpc), and with the highest pathogenicity index of H03-pCS recombinant infected groups (PI = 2.3) (Table 2). Viral RNA was detected in oropharyngeal and/or cloacal swabs in all chickens at a level comparable to H03. Antigen concentration was as high as in H03 inoculated animals in vascular endothelial cells and multiple organs (except jejunum) including lungs and brain (Fig. 4).

L01_CSIT killed 4/6 inoculated birds with MDT of 5.1 days and 2/4 contact birds died within 8 days (7 dpc) inducing the highest PI in L01-pCS recombinants infected groups (PI = 1.8) (Table 2). Viral RNA of L01_CSIT was detected in 4/6 inoculated birds at 4 dpi and 1/4 contact birds at 3 dpc without significant difference to L01 or L01_CSGE (Fig. 3). Antigen concentration was high in vascular endothelial cells but even higher in the brain, cardiac myocytes and kidneys.

L11_CSIT killed all inoculated chickens with MDT of 5.3 days and 3/4 contact birds died between 7–8 dpc. Viral RNA was detected in all inoculated birds (n = 6) and 1/4 contact birds, and the amount of excreted virus compared to L11 was significantly higher (p = 0.01) (Fig. 3). Tropism of L11_CSIT to the endothelial cells was as strong as L11_CSGE (Fig. 4) but it exhibited more antigenic load in bronchial and tracheal epithelia and lower antigen concentration in the heart and kidney.

L13_CSIT killed 5/6 inoculated chickens with MDT of 5.4 days and 3/4 of contact birds died between 6–7 dpc (Table 2). Viral RNA was detected in swabs in all inoculated chickens and 3/4 contact birds. Quantity of excreted virus was slightly higher than in L13 and L13_CSGE infected animals (Fig. 3). Viral antigen was detected in the endothelial cells of all organs, although at lower level than L13_CSGE with remarkable lower tropism to the brain, pancreas and kidney (Fig. 4).

### pCSUK conferred lower virulence, transmissibility and tissue tropism compared to pCSGE and pCSIT

On the one hand, replacement of pCSGE by pCSUK in H03 resulted in a decreased virulence of the recombinant virus. H03_CSUK killed 3/6 inoculated birds and 1/4 contact birds with MDT of 5.3 days (Table 2). Surviving chickens showed mild to moderate clinical signs and recovered at the end of the experiment. The virus was detected in swabs in all inoculated (n = 6) and 1/4 contact birds. Compared to the other H03 pCS-recombinants, about 10-fold less viral RNA was excreted (Fig. 3). Although viral antigen was detected in all vascular endothelial cells (except in trachea) of at least one bird, the amount of antigen was lower than after infection with H03 and H03_CSIT, particularly in the lung, brain and pancreas (Fig. 4).

On the other hand, insertion of pCSUK in all three LPAIVs resulted in a less drastic increase in virulence and tissue tropism than insertion of pCSGE and pCSIT. L01_CSUK killed 2 out of 6 inoculated birds with MDT of 7.5 days. All contact birds survived showing transient mild to moderate clinical signs (Table 2). Only one inoculated bird excreted virus in oropharyngeal and cloacal swabs and all contact birds tested negative. Although virus antigen was detected in vascular endothelial cells of all organs examined of at least one bird, the amount of antigen of L01_CSUK was remarkably less than L01_CSGE and L01_CSIT in endothelial cells (Fig. 4). In various organs, L01_CSUK distribution was as limited as L01_CSGE but more restricted than L01_CSIT particularly in the brain and heart.

L11_CSUK killed only 3 out of 6 inoculated birds with MDT of 5.7 days and all contact chickens survived without showing clinical signs (Table 2). Viral RNA was detected in swabs in all inoculated chickens and none of the contact birds tested positive. Amount of virus excretion was significantly higher than birds inoculated with L11 (Fig. 3). Compared to L11_CSGE, the virus was not detected in the endothelial cells of trachea, proventriculus, cecal tonsils, kidney and brain and less efficient spread, if any, to different tissues particularly lung, brain, heart, kidney and gastrointestinal tract was observed (Fig. 4).

L13_CSUK was also less virulent than L13_CSGE and L13_CSIT. Only 3 out of 6 inoculated chickens died at 3 dpi and the remaining birds recovered at the end of the experiment. None of the contact birds died, although all birds showed transient mild to severe clinical signs (Table 2). All inoculated birds excreted viruses at 4 dpi in oropharyngeal and/or cloacal swabs. Viral antigen was detected in the endothelial cells of all organs except pancreas and jejunum, although at a very low level compared to L13_CSGE (Fig. 4). In tissues of inoculated birds, particularly in brain, kidney and mucosal epithelium of gastrointestinal tract, the amount of viral antigen was very low.

### pCS motifs modulated viral replication in cell culture, the HA cleavability and plaque size independent on the HA background

**Figure 5:**
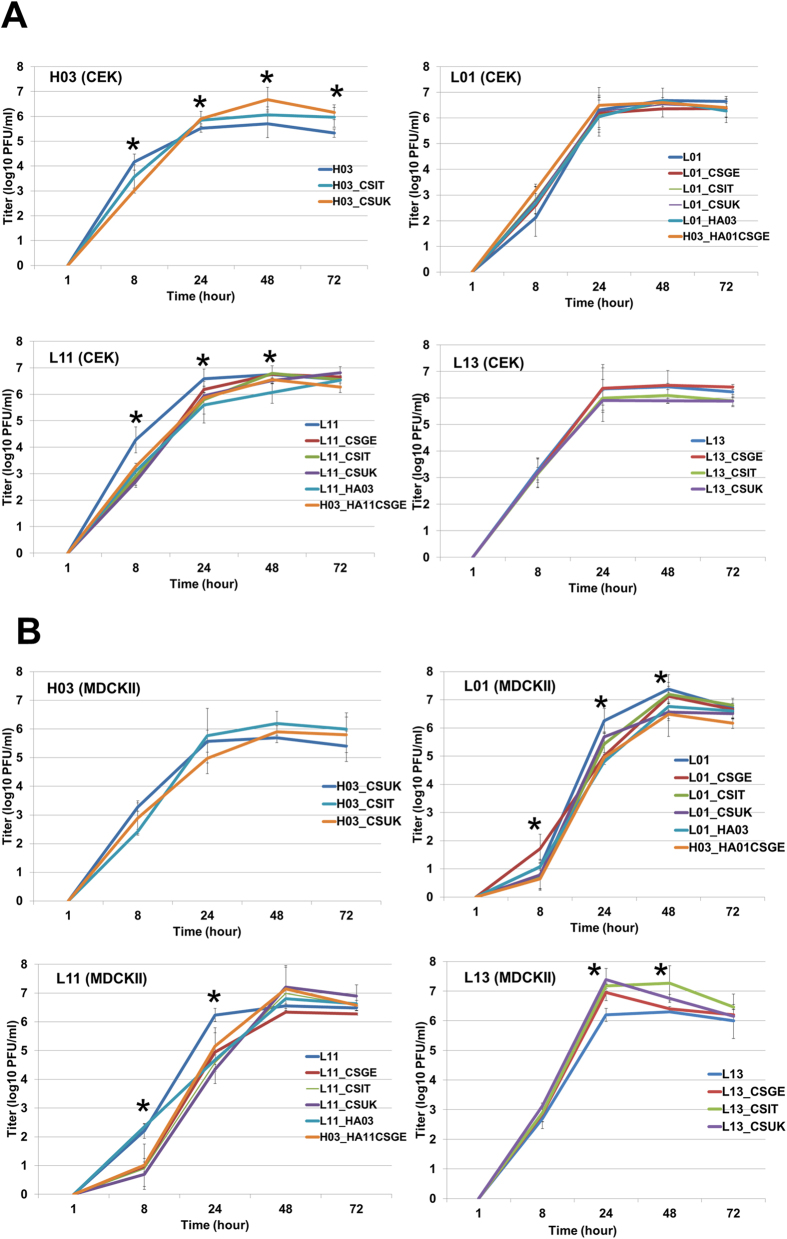
Replication kinetics of viruses in chicken embryo kidney (**A**) and Madin-Darby canine kidney cells (**B**). Shown is the titer (mean ± standard deviation of two independent experiments) of progeny virus at 1, 8, 24, 48 and 72 hours post infection in CEK (**A**) and MDCKII cells (**B**). Statistically significant differences are indicated by asterisks (p < 0.05).

All viruses in this study reached maximum titres in CEK and MDCKII cells 24–48 after infection (Fig. 5). The LPAIVs grow in the presence of trypsin to higher levels compared to LPAIVs infected cells without trypsin (Supplementary Figure S1). Therefore, the impact of different pCS motifs on replication in cell culture was compared to LPAIVs grown in the presence of trypsin. pCSGE decreased replication of L01 and L13 in MDCKII, but not in CEK cells, at 24 hpi as well as L11 in CEK at 8 hpi and MDCKII cells at 8 and 24 hpi (Fig. 5). While pCSIT did not affect replication of H03, it is significantly decreased replication of L01 in MDCKII cells at 24 hpi (Fig. 5B) and L11 in CEK and MDCKII cells at 8 and 24 hpi (Fig. 5). On the contrary, L13_CSIT replicated at higher titres than L13 at 24 and 48 hpi in MDCKII cells (Fig. 5).

In CEK, H03_CSUK replicated to higher titers than H03 at 48 and 72 hpi, but no significant difference in replication in MDCKII cells was observed (Fig. 5). In cell culture there was no significant difference in replication of L01_CSUK and L01, but L01_CSUK replicated at lower level than L01_CSGE at 8 hpi in MDCKII cells (Fig. 5B). Compared to L11, L11_CSUK replicated to significantly lower titres at 8 hpi in CEK, and at 8 and 24 hpi in MDCKII cells (Fig. 5). In CEK, L13_CSUK replicated to similar levels as the other viruses but in MDCKII cells it replicated at significantly higher levels at 24 and 48 hpi than L13 (Fig. 5).

**Figure 6:**
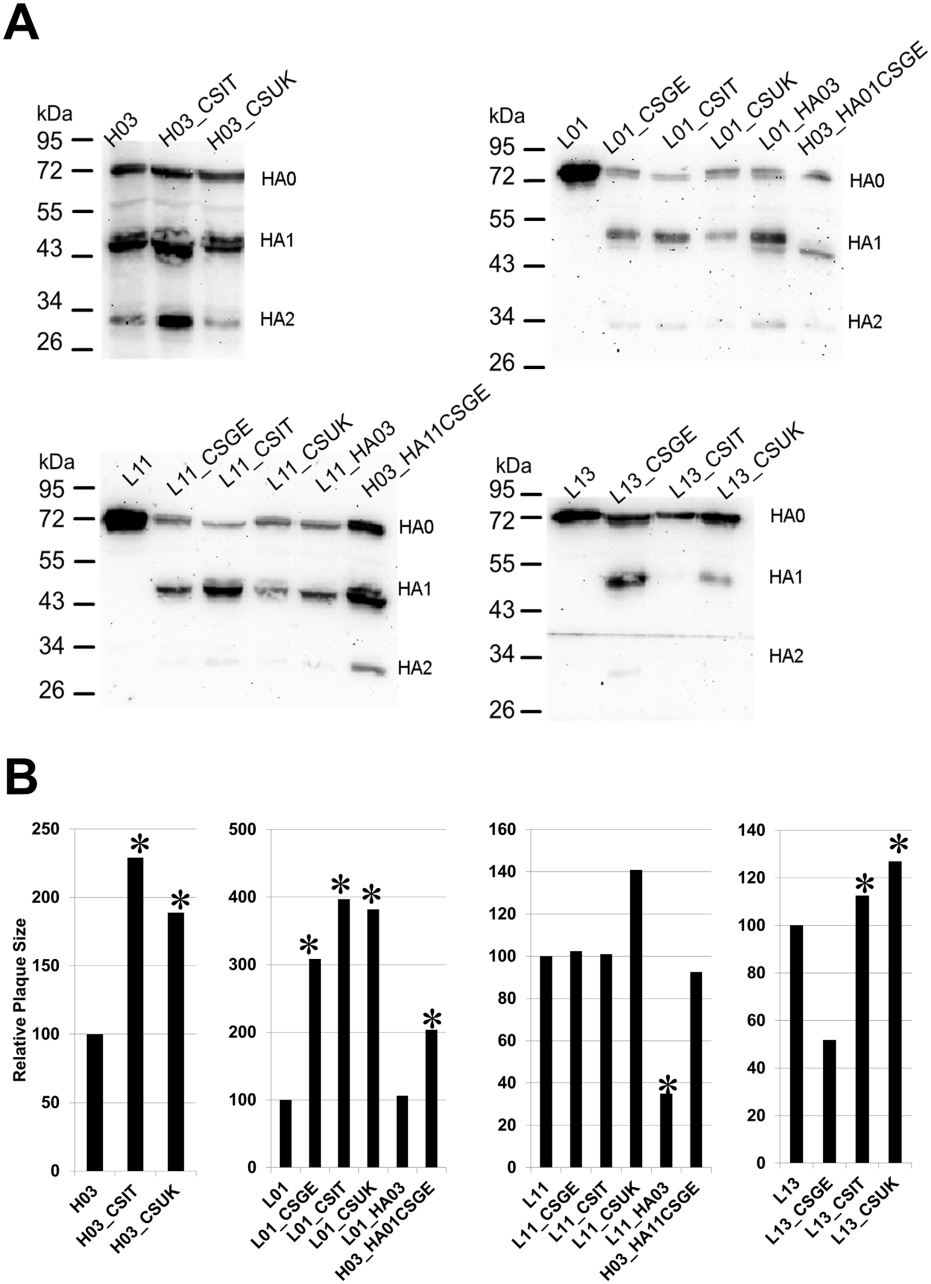
Effect of different pCS on cleavability of the HA protein (**A**) and plaque sizes (**B**). Cleavability of the HA of recombinant viruses 6 hours post infection was determined by Western Blot analyses after inoculation of CEK cells at a MOI of 1 in the absence of trypsin (**A**). Cell-to-cell spread was assessed by measuring the plaque sizes in MDCKII cells 72 hours post infection. The mean plaques sizes of the parental viruses were adjusted to 100% and the plaque sizes of recombinant viruses were given in relation to them.

**Figure 7:**
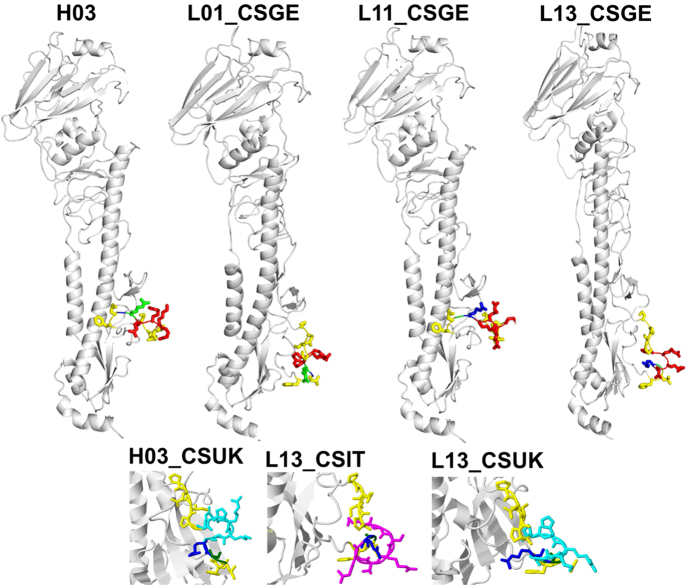
Molecular modelling of the HA protein. Predicted tertiary structure of the HA proteins of selected recombinants with pCS. Variable cleavage site motifs are illustrated: cleavage site between arginine (blue: R) and glycine (G: green), monobasic CS is depicted in yellow sticks; pCSGE in red sticks; pCSIT in magenta sticks; pCSUK in cyan sticks.

In the absence of trypsin HAs of all LPAIV strains were not cleaved in CEK using Western blot, but HA was efficiently cleaved into HA1 and HA2 subunits after insertion of pCS except for L13_CSIT (Fig. 6A). This inefficient cleavage of L13_CSIT could be due to the formation of an umbrella-like structure or a loop which apparently overlaps the CS (Fig. 7). We observed no correlation between the plaque sizes and HA cleavability. While pCSGE did no influence the plaque size of L11, it significantly increased the plaque size of L01 and decreased the plaque size of L13 (Fig. 6B). Insertion of pCSIT in H03, L01 and L13 induced significantly larger plaques than H03, L01 and L13 respectively (Fig. 6B) but no significant difference in plaque size was observed when L11_CSIT was compared to L11 and L11_CSGE (Fig. 6B). Insertion of pCSUK significantly increased the size of plaques induced by H03 and L01. Likewise, plaque size from L13_CSUK was significantly larger than from L13, L13_CSGE and L13_CSIT (Fig. 6B). Structural modelling of L13_CSUK showed that pCSUK protrudes outside the stalk domain of the HA compared to pCSGE, while the unique histidine ring of H320 in CSUK apparently shadows the CS (i.e. R^324^G^325^) (Fig. 7).

### The HA gene of HPAIV H03 was not sufficient to mediate high virulence of L01 and L11 in chickens

To study the contribution of other gene segments to virulence of H7N7, four different recombinant viruses were generated: H03 with HA gene segments of L01 (H03_HA01CSGE) or L11 (H03_HA11CSGE) after insertion of pCSGE, L01 with HA from H03 (L01_HA03) or L11 with HA from H03 (L11_HA03). After inoculation of chickens with H03_HA01CSGE all inoculated and contact birds died with MDT of 4.4 days. Likewise, H03_HA11CSGE killed all inoculated chickens with MDT of 3.5 days and 2/4 contact birds died within 4 dpc. Conversely, L01_HA03 and L11_HA03 exhibited less virulence and did not kill any inoculated birds, although birds showed transient mild to moderate clinical signs (Table 2). In addition, L01_HA03 killed 1/4 contact bird 6 dpc (Table 2).

Virus excretion in birds inoculated with H03_HA01CSGE was higher than in L01 derived viruses (including L01_HA03) indicating that other gene segments are required for increased virus excretion from inoculated birds (Fig. 3). L01_HA03 was not detected in vascular endothelial cells of any organ examined resembling L01, whereas higher amounts of H03_HA01CSGE were detected. In CEK, all viruses replicated at approximately similar levels but in MDCKII cells H03_HA01CSGE and L01_HA03 replicated at significantly lower levels at 24 and/or 48 hpi compared to L01 (Fig. 5). Plaques induced by L01_HA03 were as large as those induced by L01 and significantly smaller than those induced by H03_HA01CSGE (Fig. 6B).

Excretion of L11 was considerably increased at 4 dpi after reassortment of the HA gene segment of H03 (Fig. 3). L11_HA03 was detected in a limited number of organs, whereas H03_HA11CSGE spread widely resembling L11_CSGE. L11_HA03 was detected in the endothelial cells of heart, trachea, pancreas and liver only and in remarkably lower amount than H03_HA11CSGE. In CEK, no significant difference was observed between the level of replication of the two viruses, however both viruses replicated at significantly lower levels than L11 at 8 and 24 hpi. In MDCKII, H03_HA1361CSGE replicated to significantly lower levels than L11 and L11_HA03 at 8 hpi. Moreover, L11_HA03 and H03_HA11CSGE replicated at significantly lower levels than L11 at 24 hpi (Fig. 5). Plaques induced by L11_HA03 were significantly smaller than L11 and H03_HA11CSGE (Fig. 6B).

### Serial passages of L11 in 14-day old embryonated chicken eggs induced changes in the HA but outside the cleavage site

It has been previously reported that some H5/H7 LPAIV may shift to high virulence after passage in embryonated chicken eggs^26^,^27^. Therefore, L11 was inoculated into 14-day-old ECE for 16 serial passages. Sequence analysis of the HA gene revealed 4 mutations in the HA (3 non-synonymous in the HA1 head domain R121I, E177G and S188N and one synonymous). However, none of them was within or adjacent to the CS (i.e. the CS remained monobasic motif: PEIPKG**R**/GLF) (Fig. 7).

## Discussion

HPAIV evolves from LP precursors by stepwise mutation(s), reassortment of gene segment(s) and/or non-homologous recombination after circulation in domestic poultry^2^,^3^,^6^,^26^,^28^. Most of our knowledge on virulence of HPAIVs are driven by experiments using H5 viruses. Little is known about the virulence of H7 viruses, although they have been frequently reported in poultry causing devastating outbreaks and transmitted to humans with fatal consequences. Some of these H7 outbreaks were extensive (e.g. H7N1 in poultry in Italy in 1999–2000 and HP H7N7 in poultry and humans in the Netherlands, Belgium and Germany in 2003), while others were limited (e.g. HPAI H7N7 outbreak in poultry in the UK in 2015). Each HPAIV in these outbreaks possessed a unique pCS. In this study, we investigated the impact of different pCS motifs on virulence in chickens of four H7N7 viruses isolated in Germany.

The driving forces for the evolution of HP from LP ancestors through acquisition of a certain pCS remain poorly understood. Several mechanisms have been proposed to explain the acquisition of a pCS motif in the HA such as polymerase slippage due to secondary structure in or adjacent to the CS resulting in substitution or insertion of purine-rich arginine or lysine codons^29^,^30^ which may explain the acquisition of pCSGE whereas insertion mechanisms of additional (mostly basic) amino acids in pCSIT and pCSUK remain unknown^31^. Also, recombination of the HA with nucleoprotein^28^, matrix^32^ genes or host 28 S rRNA^33^ may result in the formation of a loop structure bulging out from the HA which probably enhances the accessibility of furin to pCS and thus increases virulence^28^,^32^,^33^. Moreover, the existence of a basic amino acid at position −4 (P4) from the CS (as seen in pCSGE, pCSIT and pCSUK) was essential for the cleavage activation of the HA, and thus virulence, of AIV^34^. In this study, pCSGE and pCSIT conferred high virulence in chickens to LPAI H7N7 viruses, wide dissemination in different organs and increased virus excretion from both inoculated and contact birds. In contrast, pCSGE did not confer high virulence to LPAIV H7N1^12^ or LPAIV H3N8^35^ and reduced transmissibility of HPAIV H7N1 in chickens (Abdelwhab *et al*., unpublished data). Likewise, pCSIT was insufficient to support full virulence of LPAIV H7N1^12^. A modification of the monobasic CS of LPAIV H7N2 to different pCS motifs modulated the virulence in chickens which varied according to the number of basic amino acids in the CS^36^. Therefore, it is likely that the tertiary structure of HA of some viruses are compatible with only certain pCS which may explain the uniqueness of each pCS in different HPAIV outbreaks.

Importantly, pCSUK complies with the minimum R-x-x-R motif for cleavage-activation by the ubiquitous furin-like proteases, increased virulence, transmissibility, virus excretion and disseminated tissue tropism in inoculated birds and enhanced cell-to-cell spread and cleavage of the HA of LPAIVs in the absence of trypsin. Nevertheless, virulence and tropism to different organs particularly lungs and brain were significantly lower than in pCSGE or pCSIT containing viruses which caused multiple organ dysfunction and high morbidity/mortality. Unfortunately, there are no data available on the virulence of the British H7N7/2015 virus in chickens. The lower virulence of pCSUK containing viruses compared to viruses containing pCSGE or pCSIT may be due to the conformational changes by the uncommon histidine (H320; position −5) in the pCS. Histidine is a unique basic amino acid changing the side chain from neutral to positive charge and, therefore, is flexible to be buried in the protein core or exposed to solvent^37^,^38^. The imidazole ring of histidine may sterically hinder the access of some activating host proteases *in vivo*. However, there was no difference *in vitro* in cleavability after insertion of pCSUK compared to pCSIT and pCSGE using Western blot. A more sensitive assay than Western blot (e.g. ELISA) may be required for quantitation of subtle differences in cleavability of HA.

While none of the pCS directly conferred high virulence to L01 and other gene segments from H03 were required as well, L11 and L13 transformed into HPAIV after acquisition of pCSGE. Moreover, lower virus excretion and restricted tropism of L01 compared to the other viruses may indicate poor adaptation to chickens and therefore further genetic changes, besides the pCS, are required for adaptation and high virulence. It is worth mentioning that L01 was isolated from a limited number of birds in a small backyard flock, whereas L11 and L13 were isolated from several commercial poultry farms causing losses of 100,000 s of birds^16^,^19^. Thus, we assume that L11 and L13 acquired virulence/adaptation markers (e.g. deletion in the NA stalk domain^39^,^40^) due to extensive and longer circulation in domestic poultry compared to L01. Nevertheless, L11 did not shift to high virulence after 16 passages in ECE and probably more passages were required as previously done^26^,^27^.

Although, it is difficult to extrapolate the *in vitro* data obtained from cell culture to those obtained after infection of chickens^41^, a remarkable difference between the *in vitro* and *in vivo* results in the current study was observed. For example, the lower spread and replication of viruses carrying pCSUK than those carrying pCSGE and pCSIT in chickens may be due to the variation in cleavage-activation in different tissues according to the presence or absence of specific proteases^42^,^43^. However, replication kinetics, and cleavability of the HA *in vitro* showed minimal differences regardless of the pCS motifs or background virus. Our *in vitro* experiments, like other studies, used kidney cells (i.e. CEK and/or MDCKII) which support the growth of nearly all AIVs probably due to the presence of matriptase^42^. Therefore, variation in cleavability of pCS motifs, especially pCSUK, using different proteolytic enzymes or different cells should be further investigated. Likewise, the use of *ex-vivo* models^41^ (e.g. tracheal organ cultures, lung lobes) in addition to cell culture may be useful in the future.

In conclusion, not all naturally occurring polybasic pCS motifs support high level virus replication in chickens, subsequent multiple organ dysfunctions and high mortality, although only minimal differences were observed in replication and cleavability in avian cell culture. Thus, Eurasian LPAIV H7N7 may shift to high virulence after acquisition of a polybasic pCS specific for the particular virus, or by reassortment introducing several gene segments from HPAIV. To avoid this conversion, swift control measures e.g. biosecurity measures, rapid elimination of infected poultry have to be executed after detection of LPAIV H7N7 in domestic poultry.

## Materials and Methods

### Viruses and cells

LPAIVs A/turkey/Germany/R11/2001 (H7N7) designated L01, A/chicken/Germany/R1361/2011 (H7N7) designated L11, and A/turkey/Germany/R534/2013 (H7N7) designated L13 as well as HPAIV A/chicken/Germany/R28/2003 (H7N7) designated H03 were obtained from the virus repository of the Friedrich-Loeffler-Institut (FLI). Cell lines were provided by the Cell Culture Collection in Veterinary Medicine of the FLI. Madin-Darby canine kidney type II (MDCKII) cells were used for virus titration and, in combination with HEK 293 T cells, for rescue of recombinant viruses. MDCKII cells, and primary chicken embryo kidney (CEK) cells were used to assess replication kinetics of different recombinant viruses.

### Virus propagation

Specific pathogen free (SPF) embryonated chickens eggs (ECE) were purchased from Lohmann Company and handled according to the standard protocol of the World Organisation for Animal Health (OIE)^44^. All wild-type and recombinant viruses with monobasic CS were handled in biosafety level 2 (BSL2) facilities, whereas all viruses with pCS were handled in BSL3+ containments at the FLI. Viruses were propagated in the allantoic sac of 9–11 days old SPF ECE according to the standard protocol^44^.

### Generation of recombinant viruses

RNA of H7N7 viruses was extracted using QIAamp Viral RNA Mini Kit (Qiagen). Recombinant viruses were generated using reverse genetics. For this purpose, all gene segments of each virus were amplified and cloned into the plasmid pHWS*ccdB* as previously described^45^. Introduction of the selected mutations into the HA gene segments of different LP and HP viruses in this study was done using site-directed mutagenesis (primer sequences are available upon request) following the QuikChange protocol (Invitrogen). All recombinant viruses and mutants were rescued in HEK 293 T/MDCKII co-culture^45^. Supernatants were then inoculated in SPF ECE for 3–5 days. Eggs were candled daily and those with dead embryos were kept in 4 °C and allantoic fluid was collected. Egg fluids with HA titre >16 (4 log_2_) were used for further investigations. To exclude any unwanted mutation and confirm the introduced genetic changes in the constructed viruses, viral RNA was extracted and analysed by Sanger sequencing of RT-PCR amplicons as described^46^.

### Plaque assay

Indicated viruses/samples were serially 10-fold diluted and MDCKII cells were incubated with virus dilutions at 37 °C/5% CO_2_. After one hour, the cells were washed twice with phosphate buffered saline (PBS) and covered by semi-solid agar in DMEM containing 4% bovine serum albumin (BSA). LPAIVs were incubated in the presence of 2 μg/mL of N-tosyl-L-phenylalanine chloromethyl ketone (TPCK)-treated trypsin (Sigma Aldrich). Plates were incubated at 37 °C for 3 days and fixed using 10% formaldehyde containing 0.1% crystal violet. Viral titres were expressed as plaque forming units per mL (PFU/ml). Moreover, plaque sizes were measured by microscopy (Eclipse Ti-S with the software NIS-Elements, version 4.0; Nikon). The mean plaque size for each recombinant virus (about 50 plaques each) was expressed as the percentage of the mean plaque size of the respective parental reverse genetic wild-type virus in the presence of trypsin.

### Replication kinetics

CEK and MDCKII cells were infected at a multiplicity of infection (MOI) of 0.001. After one hour, the inoculum was removed, the cells were incubated with citric acid buffer (pH 3.0) for two minutes and then washed with PBS. Cells were covered by MEM containing 0.2% BSA (Sigma) and incubated at 37 °C/5% CO_2_. Infected cells with LPAIVs were grown in the presence or absence of trypsin. After 1, 8, 24, 48 and 72 hours, the cells and supernatant were harvested and stored at −80 °C. Progeny viruses titres were determined by plaque assay. The average of two experiments and standard deviations were calculated.

### Animal experiments

All animal experiments in this study were carried out in the BSL3+ animal facilities of the FLI following the German Regulations for Animal Welfare after approval by the authorized ethics committee of the State Office of Agriculture, Food Safety, and Fishery in Mecklenburg – Western Pomerania (LALLF M-V). All challenge experiments were approved by the commissioner for animal welfare at the FLI representing the Institutional Animal Care and Use Committee (IACUC).

Animal experiments were performed using 6- to 8-week-old white leghorn chickens. SPF eggs were purchased from Lohman Animal Health and incubated at the animal quarantine facilities of the FLI until hatch. Chickens were divided to separate groups, 9–10 each, where 6 birds were inoculated using 10^5 ^PFU/bird of each virus in 0.2 ml through the oculo-nasal route (about 0.1 ml in each side). One day post inoculation (dpi), 3–4 birds were added to each inoculated group to assess transmissibility of the viruses.

Birds were observed daily for clinical signs and/or mortality for 10 days. Clinical scoring was performed according to standard protocols^12^. Briefly, healthy birds were given score (0), sick birds showing one clinical sign (e.g. ruffled feather, diarrhoea, respiratory disorders, nervous manifestation) were given score (1), severely sick birds showing more than one clinical sign were given score (2) and dead birds were given score (3). Severely sick birds were euthanized and scored (3) on the next observation day. The arithmetic mean of clinical signs was calculated for all chickens. The pathogenicity index (PI) was calculated as the sum of the daily arithmetic mean values divided by 10 (the number of observation days) as previously done^12^. The PI for each virus ranged from 0 (avirulent) to 3 (highly virulent). Clinical examination was done by two expert veterinarians; one examined a blinded experiment.

Swab samples were collected at 2, 4, 6, 8 and 10 days from oropharynx and cloaca of all surviving chickens. Viral excretion was determined using generic real-time reverse-transcription polymerase chain reaction (RT-qPCR) targeting the matrix gene after automatic extraction of viral RNA using NucleoSpin 8/96 PCR Clean-up Core Kit (Macherey & Nagel GmbH) according to the manufacturer instructions. Standard curves were run in each RT-qPCR using serial dilutions of an H7N7 AIV of known PFU titre and standard curves were used to extrapolate Ct-values to PFU/ml. In this setting, the cut-off value of Ct 35 represents a calculated detection limit of 6.7 PFU/ml. Moreover, viral RNA was extracted from swabs obtained from inoculated birds 4 dpi to assess possible adaptive genetic changes during replication *in vivo*. Serial passages of L11 in 14 day-old ECE SPF were done to assess the possibility for the emergence of an HP phenotype. After 16 passages, chorioallantoic fluid was collected and the viral RNA was extracted. The HA gene was amplified and sequenced.

### Histopathology and immunohistochemistry

Samples from trachea, lungs, heart, liver, pancreas, kidneys, thymus, spleen, proventriculus, gizzard, duodenum, jejunum, cecum, bursa of Fabricius, and brain from two inoculated birds of each group were collected at 4 dpi. Samples were immediately fixed in 10% neutral buffered formalin, processed, embedded in paraffin wax, sectioned at 2–4 μm, stained with hematoxylin and eosin, and screened for histopathological changes. Immunohistochemical examination of consecutive sections was performed with the avidin–biotin–peroxidase complex method (Vector Laboratories) using a primary polyclonal rabbit anti-NP antibody (1:750) and a secondary biotinylated goat anti-rabbit IgG1 (Vector Laboratories) antibody (1:200) as described^47^. The concentration of nucleoprotein antigen was semi quantitatively assessed by scoring on a 0 to 4 severity scale for tissues: 0 = negative; 1 = single cells, 2 = scattered foci, 3 = numerous foci, 4 = coalescing foci or diffuse and on a scale of 0 to 3 for endothelium: 0 = negative; 1 = single blood vessel, 2 = multiple blood vessels, 3 = diffuse as done before^48^.

### Western Blot

Cleavability of HA was investigated after inoculation of CEK cells at a MOI of 1. After 6 hours at 37 °C, cells were harvested and proteins were separated by discontinuous sodium dodecyl sulfate-10% polyacrylamide gel electrophoresis (SDS-PAGE). Transfer to nitrocellulose membranes and incubation of the blots were performed as described^12^. The HA protein was detected using a monospecific antiserum against H7 HA at a dilution of 1:1000 and peroxidase-conjugated chicken IgY-specific goat IgG (Dianova) at a dilution of 1:20000. Immunodetection was achieved by chemiluminescence using Supersignal West Pico chemiluminescent substrate kit (ThermoScientific) and images were captured using a Bio-Rad VersaDoc Imaging System and Quantity One software.

### Sequence analysis and molecular modelling

The genomes of all viruses used in this study were amplified and subjected to Sanger sequencing according to the standard methods. Analyses of the final genome sequence were conducted in Geneious software suite v.8.1.3 (Biomatters). Gene sequences generated in this study (n = 32) were submitted to the Global Initiative on Sharing All Influenza Data (GISAID) and assigned accession number: EPI772731 to EPI772762. Furthermore, HA gene sequences (n = 1884) of all H7 influenza viruses (until 16-03-2016) were retrieved from GenBank and GISAID. All sequences were aligned by MAFFT^49^, BioEdit^50^ and further edited manually. Phylogenetic trees were generated by IQ-TREE and MrBayes after selection of the best fit model using Topali v2^51^ and further edited using FigTree and Inkscape free software. HA of wild-type viruses was used for the prediction of different conformational changes in the tertiary structure using SWISS MODEL (http://swissmodel.expasy.org/), and edited by Geneious v.8.1.3. H7 numbering was used to identify amino acid positions of mature HA after removal of the signal peptide. The CS regions on the 3D structure are shown as ^315^PEIPKGR*GLF^324^ for monobasic CS of L01, L11 and L13, ^315^PEIPKRRRR*GLF^326^ for CSGE, ^315^PEIPKGSRVRR*GLF^328^ for CSIT and ^315^PEIPRHRKGR*GLF^327^ for CSUK.

### Statistics

ANOVAs with post hoc Tukey tests were utilized to compare replication kinetics. Statistical differences for amount of virus excretion, pathogenicity index and plaque size were evaluated using Kruskal-Wallis tests and Mann-Whitney-Wilcoxon tests with Benjamini-Hochberg correction. Significant differences of clinical scoring between groups were assessed by comparing the mean clinical score per bird during a 10 days observation period. A p-value of p < 0.05 was considered to be significant and all analysis was done using R version 3.2.1 from the R Foundation for Statistical Computing available at the R-project website (http://www.r-project.org).

## Additional Information

**How to cite this article**: Abdelwhab, E. M. *et al*. Composition of the Hemagglutinin Polybasic Proteolytic Cleavage Motif Mediates Variable Virulence of H7N7 Avian Influenza Viruses. *Sci. Rep.* **6**, 39505; doi: 10.1038/srep39505 (2016).

**Publisher's note:** Springer Nature remains neutral with regard to jurisdictional claims in published maps and institutional affiliations.

## Supplementary Material

Supplementary Information

## Figures and Tables

**Table 1:** Recombinant viruses generated in this study.

No.	Virus	Abbreviation	Mutation/reassortment	Cleavage site
1	H03 A/chicken/Germany/R28/2003 (H7N7)	H03	Recombinant HPAIV	PEIPK--**RRRR**/GLF
2		H03_CSIT	H03 carrying pCS from A/chicken/Italy/445/1999 (H7N1)	PEIPKG**SRVRR**/GLF
3		H03_CSUK	H03 carrying pCS from A/chicken/England/26352/2015 (H7N7)	PEIP**R**-**HRKGR**/GLF
4	L01 A/turkey/Germany/R11/2001 (H7N7)	L01	Recombinant LPAIV	PEIPKG----**R**/GLF
5		L01_CSGE	L01 carrying pCS from A/chicken/Germany/R28/2003 (H7N7)	PEIPK--**RRRR**/GLF
6		L01_CSIT	L01 carrying pCS from A/chicken/Italy/445/1999 (H7N1)	PEIPKG**SRVRR**/GLF
7		L01_CSUK	L01 carrying pCS from A/chicken/England/26352/2015 (H7N7)	PEIP**R**-**HRKGR**/GLF
8		L01_HA03	L01 carrying HA from A/chicken/Germany/R28/2003 (H7N7)	PEIPK--**RRRR**/GLF
9		H03_HA01CSGE	H03 carrying HA from A/turkey/Germany/R11/2001 (H7N7) with pCS from H03	PEIPK--**RRRR**/GLF
10	L11 A/chicken/Germany/R1361/2011 (H7N7)	L11	Recombinant LPAIV	PEIPKG----**R**/GLF
11		L11_CSGE	L11 carrying pCS from A/chicken/Germany/R28/2003 (H7N7)	PEIPK--**RRRR**/GLF
12		L11_CSIT	L11 carrying pCS from A/chicken/Italy/445/1999 (H7N1)	PEIPKG**SRVRR**/GLF
13		L11_CSUK	L11 carrying pCS from A/chicken/England/26352/2015 (H7N7)	PEIP**R**-**HRKGR**/GLF
14		L11_HA03	L11 carrying HA from A/chicken/Germany/R28/2003 (H7N7)	PEIPK--**RRRR**/GLF
15		H03_HA11CSGE	H03 carrying HA from A/turkey/Germany/R1361/2011 (H7N7) with pCS from H03	PEIPK--**RRRR**/GLF
16	L13 A/turkey/Germany/R534/2013 (H7N7)	L13	Recombinant LPAIV	PEIPKG----**R**/GLF
17		L13_CSGE	L13 carrying pCS from A/chicken/Germany/R28/2003 (H7N7)	PEIPK--**RRRR**/GLF
18		L13_CSIT	L13 carrying pCS from A/chicken/Italy/445/1999 (H7N1)	PEIPKG**SRVRR**/GLF
19		L13_CSUK	L13 carrying pCS from A/chicken/England/26352/2015 (H7N7)	PEIP**R**-**HRKGR**/GLF

Viruses were arranged in the table according to the experimental settings *in vivo* and *in vitro*. (i.e. viruses no. 1–3, 4–9, 10–15 and 16–19 were examined in 4 independent experiments). Amino acid sequences at the proteolytic cleavage site of H03 were determined in this study, which was identical to the vast majority of the parent Dutch H7N7 viruses. Sequence of CSIT was obtained from HP A/chicken/Italy/445/1999 (H7N1) used in our previous study^12^ and sequence of CSUK was obtained from A/chicken/England/26352/2015 (H7N7) gratefully deposited by Ian Brown and colleagues from Animal and Plant Health Agency (APHA), Weybridge, United Kingdom. Position -4 in the cleavage site is underlined.

**Table 2:** Clinical examination and pathogenicity index (PI) of inoculated and contact birds.

No.	Virus	Abbreviation	PI	Mortality (MDT; range of mortality per day after inoculation or after contact)		Shedding at 4dpi (No. of positive/Total examined)	
				Infected	Contact	Oral	Cloacal
1	H03 A/chicken/Germany/R28/2003 (H7N7)	H03	2.1	5/6 (4.2; 4–5)	3/4 (5–7)	4/6	4/6
2		H03_CSIT	2.3	6/6 (4.2; 3–5)	4/4 (6–8)	5/6	5/6
3		H03_CSUK	1.4	3/6 (5.3; 5–6)	1/4 (6)	6/6	6/6
4	L01 A/turkey/Germany/R11/2001 (H7N7)	L01	0.3	0/6 (n.a.)	0/4 (n.a.)	3/6	0/6
5		L01_CSGE	1.2	2/6 (6.5; 4–9)	2/4 (7–8)	3/6	4/6
6		L01_CSIT	1.8	4/6 (5.1; 3–5)	2/4 (6–7)	3/6	2/6
7		L01_CSUK	0.9	2/6 (7.5; 5–10)	0/4 (n.a.)	1/6	1/6
8		L01_HA03	0.6	0/4 (n.a.)	1/4 (6)	4/6	2/6
9		H03_HA01CSGE	2.4	6/6 (4.4; 3–5)	4/4 (6–9)	3/3	3/3
10	L11 A/chicken/Germany/R1361/2011 (H7N7)	L11	0.2	0/6 (n.a.)	0/4 (n.a.)	5/6	3/6
11		L11_CSGE	2.4	6/6 (4.2; 4–5)	4/4 (7–8)	5/5	5/5
12		L11_CSIT	2.1	6/6 (5.3; 4–7)	3/4 (7–8)	6/6	6/6
13		L11_CSUK	1.2	3/6 (5.7; 5–7)	0/3 (n.a.)	6/6	6/6
14		L11_HA03	0.4	0/4 (n.a.)	0/4 (n.a.)	3/3	3/3
15		H03_HA11CSGE	2.5	6/6 (3.5; 3–4)	2/4 (4)	6/6	6/6
16	L13 A/turkey/Germany/R534/2013 (H7N7)	L13	0.2	0/6 (n.a.)	0/4 (n.a.)	4/6	2/6
17		L13_CSGE	2.5	6/6 (3; 3)	4/4 (4–5)	5/6	3/6
18		L13_CSIT	1.9	5/6 (5.4; 5–7)	3/4 (6–7)	5/6	6/6
19		L13_CSUK	1.4	3/6 (5; 5)	0/4 (n.a.)	5/6	5/6

Viruses were arranged in the table according to the experimental settings *in vivo* and *in vitro*.

MDT = Mean death time, n.a. = not applicable.

The pathogenicity index (PI) was calculated for each group as the sum of the daily arithmetic mean values divided by 10; the number of observation days. Virus shedding in swab samples from inoculated chickens was estimated by a generic real-time reverse transcription polymerase chain reaction assay.
