# Supplementary material for: Composition of the Hemagglutinin Polybasic Proteolytic Cleavage Motif Mediates Variable Virulence of H7N7 Avian Influenza Viruses
Source: Sci Rep. 2016 Dec 22;6:39505. doi: 10.1038/srep39505 (PMC5177941; doi:10.1038/srep39505)
Supplement: Supplementary Information [file srep39505-s1.pdf]

Composition of the Hemagglutinin Polybasic Proteolytic Cleavage Motif Mediates Variable Virulence of H7N7 Avian Influenza Viruses

El-Sayed M. Abdelwhab[1]#, Jutta Veits[1], Reiner Ulrich[2], Elisa Kasbohm[3], Jens P. Teifke[2] and Thomas C. Mettenleiter[1]

[1] Institute of Molecular Virology and Cell Biology, Friedrich-Loeffler-Institut, Federal Research Institute for Animal Health, Greifswald-Insel Riems, Germany

[2] Department of Experimental Animal Facilities and Biorisk Management, Friedrich-Loeffler-Institut, Federal Research Institute for Animal Health, Greifswald-Insel Riems, Germany

[3] Institute of Epidemiology, Friedrich-Loeffler-Institut, Federal Research Institute for Animal Health, Greifswald-Insel Riems, Germany

Running Title: Virulence of Eurasian H7N7 in chickens

# Corresponding author:

Mailing Address: Friedrich-Loeffler-Institut,

Suedufer 10, 17493 Greifswald-Insel Riems, Germany

Tel.: +49 38351 7 1139

Fax: +49 38351 7 1188

E-mail: sayed.abdel-whab@fli.bund.de; sayedabdelwhab@yahoo.com

Supplementary Figure S1: Replication of recombinant low pathogenic H7N7 avian influenza viruses generated in this study in the presence or absence of trypsin.

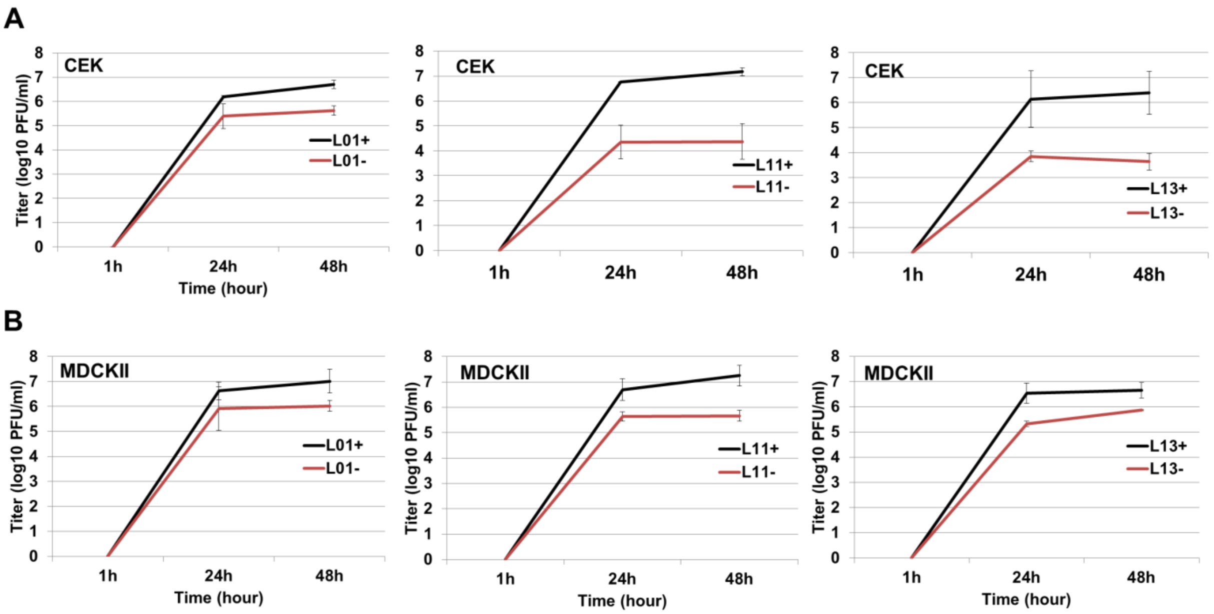

CEK and MDCKII cells were infected at MOI of 0.001 for 1, 24 and 48 hours in the presence or absence of trypsin. Virus titration was done in MDCKII in the presence of trypsin. The test was done in two independent replicates and shown are the average and standard deviation.
